# Predicting Early Intrahepatic Recurrence of Hepatocellular Carcinoma after Microwave Ablation Using SELDI-TOF Proteomic Signature

**DOI:** 10.1371/journal.pone.0082448

**Published:** 2013-12-13

**Authors:** Xiao-lin Cao, Hua Li, Xiao-ling Yu, Ping Liang, Bao-wei Dong, Jin Fan, Meng Li, Fang-yi Liu

**Affiliations:** 1 Department of Interventional Ultrasound, General Hospital of People's Liberation Army, Beijing, China; 2 Department of Ultrasound, Southern Building Clinic Division, General Hospital of People's Liberation Army, Beijing, China; 3 Department of Ultrasound, the 306^th^ Hospital of Chinese People's Liberation Army, Beijing, China; West German Cancer Center, Germany

## Abstract

**Background/Aims:**

Despite great progress in the treatment of hepatocellular carcinoma (HCC) over the last-decade, intrahepatic recurrence is still the most frequent serious adverse event after all the treatments including microwave ablation. This study aimed to predict early recurrence of HCC after microwave ablation using serum proteomic signature.

**Methods:**

After curative microwave ablation of HCC, 86 patients were followed-up for 1 year. Serum samples were collected before microwave ablation. The mass spectra of proteins were generated using surface-enhanced laser desorption/ionization time-of-flight mass spectrometry (SELDI-TOF MS). Serum samples from 50 patients were randomly selected as a training set and for biomarkers discovery and model development. The remaining serum samples were categorized for validation of the algorithm.

**Results:**

According to preablation serum protein profiling obtained from the 50 HCC samples in the training set, nine significant differentially-expressed proteins were detected in the serum samples between recurrent and non-recurrent patients. Decision classification tree combined with three candidate proteins with m/z values of 7787, 6858 and 6646 was produced using Biomarker Patterns Software with sensitivity of 85.7% and specificity of 88.9% in the training set. When the SELDI marker pattern was tested with the blinded testing set, it yielded a sensitivity of 80.0%, a specificity of 88.5% and a positive predictive value of 86.1%.

**Conclusions:**

Differentially-expressed protein peaks in preablation serum screened by SELDI are associated with prognosis of HCC. The decision classification tree is a potential tool in predicting early intrahepatic recurrence in HCC patients after microwave ablation.

## Introduction

Hepatocellular carcinoma (HCC) is an aggressive cancer with an overall poor prognosis. Known to be the fifth most prevalent cancer worldwide, it is the third most common cause of cancer-related death; 81% of cases occur in the less developed countries and 54% in China [1].

A major obstacle in HCC treatment is intrahepatic recurrence, which is high in patients who undergo hepatic resection [2], [3]. Microwave ablation (MWA), as a minimally invasive management technique, has been developed and improved greatly in the last few years. It has been widely used as an effective approach to HCC treatment in China because of its minimal damage to liver function, convenient manipulation, reduced complications, and lower mortality [4]. Despite complete ablation of the tumor, intrahepatic recurrence limits the potential therapeutic effect on HCC [5]. Therefore, it is urgent to find novel methods that provide valuable information for screening and early predicting recurrence of HCC.

Although some traditional serum biomarkers play a role in clinical diagnosis and surveillance of tumor recurrence, their contribution is limited, partly because of low sensitivity and specificity. Development of high throughput proteomics technology provides a new pathway to large-scale screening and identification of biomarkers in body fluid (serum, plasma, urine, *etc*.) and tissue [6], [7]. In recent years, an innovative proteomic technology, serum surface-enhanced laser desorption/ionization time-of-flight mass spectrometry (SELDI-TOF MS), has overcome many of the limitations of two-dimensional electrophoresis [8], [9]. SELDI is a type of mass spectrometry that is useful in high throughput proteomic fingerprinting of complex biological specimens such as serum that uses on-chip protein fractionation coupled to time-of-flight separation. It can detect multiple protein changes simultaneously with high sensitivity and specificity [10]–[12].

Recently, SELDI-TOF-MS has been successfully applied to the identification of serum biomarkers for the detection of liver, ovarian, pancreatic, breast and prostate cancer patients [13]–[20], but has not been used specifically to predict recurrence in diseases such as breast, prostate, and liver cancer so far. The current study was designed to investigate the application of serum SELDI protein profiling in prediction of early intrahepatic recurrence of HCC.

## Materials and Methods

### Patients and Samples

Our study was approved by the Ethics Committee of Chinese PLA General Hospital, and all study procedures were conducted therein. All study participants provided written informed consent, which was approved by the Ethics Committee of Chinese PLA General Hospital. Patients enrolled in the study fulfilled the following criteria: 1) single HCC nodule (≤5 cm); 2) no portal thrombosis or extrahepatic metastases; 3) Child-Pugh class A or B; 4) histologically proven HCC; and 5) no treatment undertaken before MWA. From March 2006 to March 2008, 86 eligible HBV-related HCC patients were enrolled.

Blood samples from 86 patients with HCC were obtained before initiation of treatment and allowed to clot at room temperature for 30 minutes. Serum was separated by centrifugation at 1500×g for 15 minutes. All samples were divided into 20 µl aliquots and frozen at −80°C until analysis. Each sample used for proteomic profiling (SELDI ProteinChip analysis) had not been thawed more than once. All samples were randomly assigned to a training set (50 serum samples) and a testing set (36 serum samples).

### Microwave Ablation Procedure

All treatments were performed in our institution and were carried out under sonographic guidance. Tumors were treated by means of microwave ablation using a KY-2000 microwave applicator (Kangyou Medical, China) with a frequency of 2450 MHz delivering a maximum power of 100 W through two 15-gauge internally-cooled antennae [4]. Single antenna or multiple antennas were used under sonographic guidance, depending on the tumor size. A detailed protocol was determined for each patient on an individual basis before treatment, inclusive of the placement of the antennas, power output setting, emission time, and appropriate approach. In general, single antenna was used for tumor less than 1.7 cm in diameter, while multiple antennas were required if tumor was larger or equal to 1.7 cm. An output setting of 60 W for 300 seconds was routinely used during ablations. A thermal monitoring system was applied during treatment. With sonographic guidance, one to three thermal couples were placed at different sites 0.5 cm outside the tumor to monitor temperature throughout the procedure. If the measured temperature did not reach 60°C by the end of 300 seconds or could not remain constant at 54°C for at least 3 minutes, a prolonged emission time was required depending on the temperature, which was monitored dynamically. General anesthesia was given to all patients by using a combination of two anesthetics, propofol (Diprivan; Zeneca Pharmaceuticals, Wilmington, DE, USA) and ketamine (Shuanghe Pharmaceuticals, Beijing, China) via the peripheral veins.

### Follow-up

The follow-up period was calculated starting from the end of microwave ablation for all patients. The patients were followed-up regularly for the detection of recurrence by ultrasonography, contrast enhanced ultrasound (CEUS) and/or contrast enhanced computed tomography/contrast enhanced magnetic resonance imaging (CECT/CE-MRI) 1 month after MWA ablation treatment and then every 3 months. The therapeutic response was considered accomplished when CEUS and CECT/CE-MRI findings showed no areas of contrast material enhancement in the lesion. All patients were followed-up for 1 year until April 2009. We defined curative MWA as complete ablation of the tumor with no residual tumors, as indicated by CEUS and CECT/CE-MRI scans at 1 year after MWA.

In the 1 year follow-up period, no local tumor regrowth was observed in any of the patients enrolled in the study. However, in 24 patients (14 in the training set, 10 in the testing set), there was recurrence in another segment of liver. Recurrence was diagnosed when detected by CEUS or CECT/CE-MRI and identified by biopsy and imaging findings. There was no statistical difference between the two patient groups for sex, age, tumor size, tumor location, and Child-Pugh classification, but preablation serum α-fetoprotein (AFP) level differed significantly (Table 1).

**Table 1:** Characteristics of 86 patients treated with MWA.

Variables	Definitions	Non-recurrence(n = 62)	Recurrence(n = 24)	*P Value*
Sex	Male	51	22	0.449
	Female	11	2	
Age (yr)		57.9±9.8	54.7±8.6	0.164
Child-Pugh classification	A	59	22	0.914
	B	3	2	
Tumor location	Left lobe	11	5	0.983
	Right lobe	51	19	
Maximum tumor size	≤3 cm	45	16	0.588
	3∼5 cm	17	8	
Preablation serum AFP Level (ng/ml)	AFP≤20	43	11	0.043
	AFP>20	19	13	

### SELDI Processing of Serum Samples

We used CM10 ProteinChip arrays incorporated with a carboxylate group that acts as a weak cation exchanger for analysis. Chips were rinsed with ultrapure water and inserted into the bioprocessor (Ciphergen Biosystems, Inc.). Serum samples were analyzed with the SELDI ProteinChip system (Ciphergen Biosystems) to obtain a quantitative proteomic profile. Briefly, 10 µl of each sample was denatured by addition of 20 µl of U9 solution (9 mol/L urea, 2% CHAPS, 50 mmol/L Tris-HCl, pH 9.0), then diluted with binding buffer (50 mmol/L sodium acetate, pH 4.0) to give a final dilution of 40-fold. CM10 ProteinChip arrays were pre-equilibrated twice with 200 µl of the binding buffer at a speed of 400 rpm for 5 minutes, after which 100 µl of the diluted sample was applied to the ProteinChip arrays and incubated with shaking at room temperature for 60 minutes. Then, each array was washed twice with the binding buffer followed by rinsing once with deionized water. After air drying, each array was treated twice with 0.5 µl sinapinic acid solution and allowed to air-dry.

### Processing of SELDI Data

The ProteinChip arrays were read on the ProteinChip PBS II reader of the ProteinChip Biomarker System to measure the masses and intensities of the protein peaks. The starting laser intensity was set to 225, with a detector sensitivity of 9. SELDI acquisition parameters were set to 24, delta to 5, transients per to 5, and ending position to 84. Spectra between 1,000 and 30,000 m/z were selected for analysis. Smaller masses were not analyzed, because they were considered to be artifacts of energy absorbing molecules. Peak detection was performed using Ciphergen ProteinChip Software, version 3.1.1 (Ciphergen Biosystems).

Based on normalized intensity levels of the peaks from the SELDI protein expression profile, a decision tree was constructed from the training set. The training set consisted of spectral data from 14 HCC patients with recurrence and 36 non-recurrent HCC patients. The classification tree began at the roof node, and split the data into two nodes using one rule at a time in the form of a question. The process of splitting was continued until the terminal nodes were produced. Multiple classification trees were generated using this process, and the best performing tree was chosen for testing. The discriminatory ability of the classification algorithm was then challenged with a blinded testing set consisting of spectral data from 10 additional HCC patients with recurrence and 26 additional non-recurrent HCC patients. For each sample, the intensity values for each peak within the 1,000–30,000 m/z range were inputted into the Biomarker Patterns Software (Ciphergen Biosystems) and classified according to the tree analysis described above. Then we modeled the results by mathematical equations.

### Statistical Analysis

In the Biomarker Wizard mode, comparison of the normalized peak intensity of each peak detected in sera between recurrent HCC patients and non-recurrent HCC patients was determined with the Student *t*-test. *P* values of <0.05 were considered statistically significant. Specificity was calculated as the ratio of the number of negative samples correctly classified to the total number of true negative samples. Sensitivity was calculated as the ratio of the number of correctly classified positive samples to the total number of true positive samples.

## Results

### Serum Protein Peaks Associated with Recurrence of HCC

**Figure 1:**
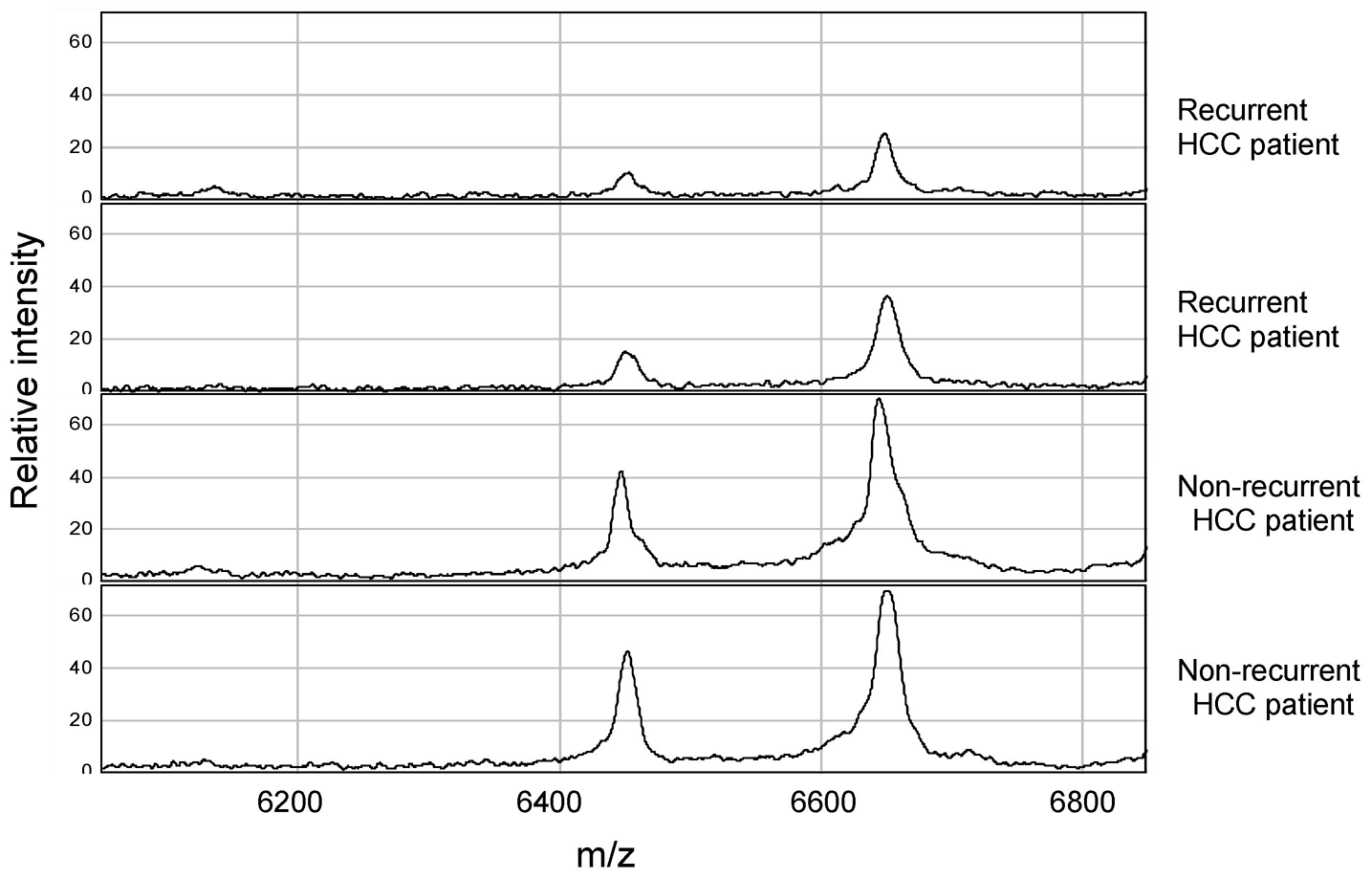
Representative mass spectra ranging from 6200–6800 m/z from the sera of recurrent and non-recurrent patients. The horizontal axis indicates protein mass to charge (m/z), and the longitudinal axis designates the relative intensity. Peaks of 6449 and 6646 m/z are shown. The box indicates that peaks of 6449 and 6646 m/z were overexpressed in the non-recurrent group compared with the recurrent group.

Eighty-six serum samples were assayed by SELDI mass spectrometry. From a total of 86 serum samples, 50 serum samples (36 non-recurrent and 14 recurrent) were randomly selected to form the learning set and 36 serum samples (26 non-recurrent and 10 recurrent) to form the blinded testing set for the algorithm. The analysis of protein spectra from the training set identified 87 signal peak protein clusters within the 1,000 to 30,000 m/z range. Nine protein peaks differed significantly. One protein peak at 5170 m/z was over-expressed, whereas nine protein peaks with m/z values of 4100, 6449, 6646, 6858, 7787, 8482, 8583, and 8933 were down-regulated significantly in sera from recurrent patients in comparison with non-recurrent patients. Figure 1 shows representative protein spectra from non-recurrent and recurrent patients. The mean amplitudes of the peaks for recurrent and non-recurrent HCC patients are shown in Table 2.

**Table 2:** Discriminatory peaks and mean values between recurrent and non-recurrent patients in training set.

M/Z	Non-recurrence (n = 36)	Recurrence (n = 14)	*P* Value
6858	11.298±3.465	7.860±1.949	0.0001
7787	7.295±4.710	3.986±1.888	0.0009
6646	52.296±11.492	41.489±9.392	0.0030
6449	28.330±10.068	20.680±7.752	0.0073
8583	20.831±8.600	15.620±5.746	0.0179
4100	22.180±9.720	15.626±8.634	0.0278
8933	10.360±3.243	8.517±2.329	0.0323
8482	3.572±1.434	2.607±1.415	0.0371
5170	1.067±0.853	2.301±2.039	0.0447

### Structure of the Decision Tree

**Figure 2:**
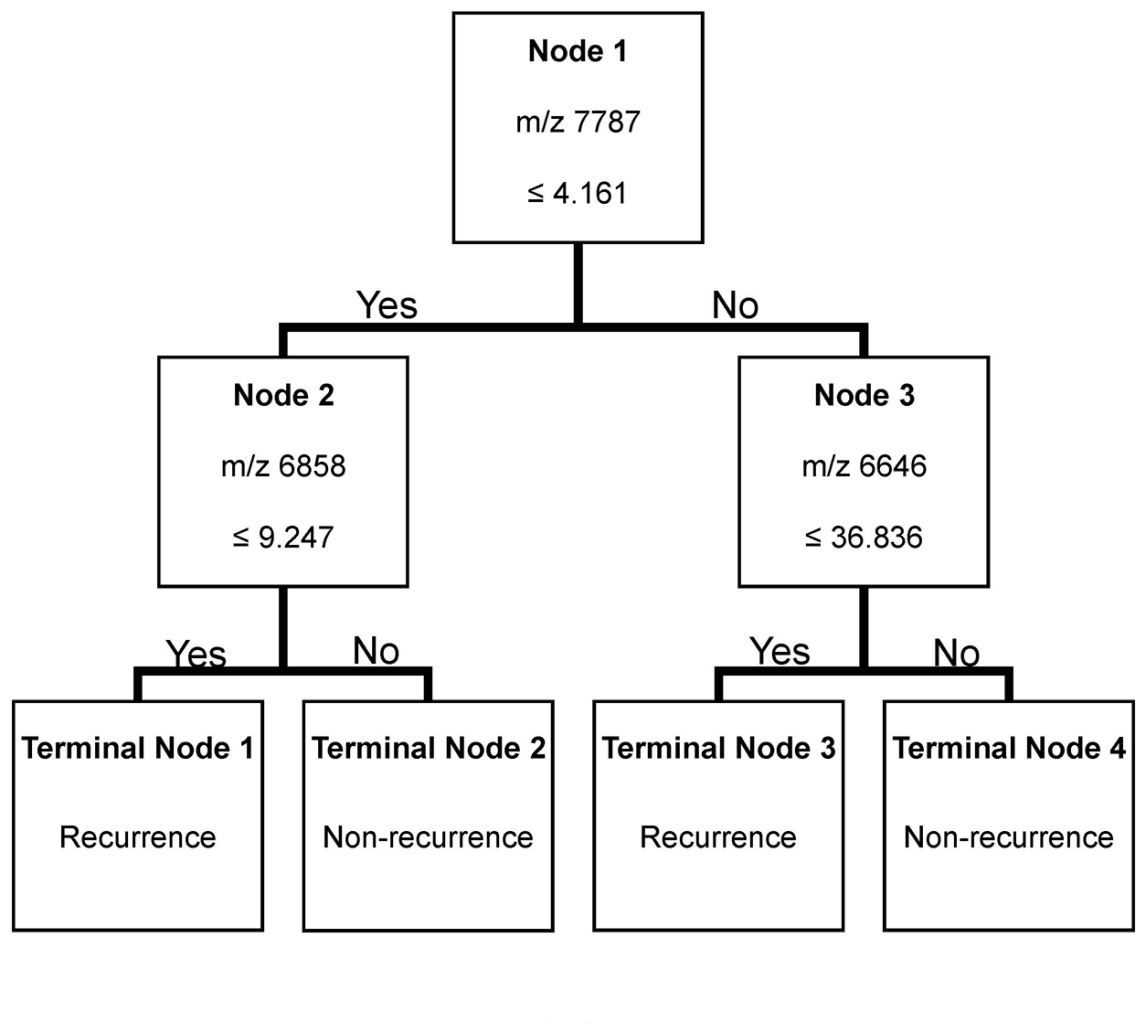
Classification of recurrence and non-recurrence samples of 56 HCC patients by the decision classification tree algorithm in the training set. The left branch node after the first layer (7787 m/z) includes cases of peak intensity less than or equal to 4.161, the right node with those greater than 4.161. The cutoff points for 6858 and 6646 m/z were 9.247 and 36.836, respectively. The decision tree was constructed to correctly classify 85.7% of the recurrent patients in the training set.

The decision tree is a flowchart-like tree structure that repeatedly splits data sets into subsets in accordance with the given recurrence versus non-recurrence classification task. Each classifier, a simple rule applied to each patient, queries only one protein peak. Using the normalized peak intensities of these 87 signal clusters, we constructed and evaluated the decision tree using the training set. The decision classification tree combined with three candidate protein peaks with m/z values of 7787, 6858 and 6646 were produced by the Biomarker Patten Software with a sensitivity of 85.7% and a specificity of 88.9%. The decision tree generated using a combination of these three protein peaks correctly classified 88.0% (44 of 50) of sera samples (Figure 2, Table 3). We modeled the results on the basis of the decision classification tree by mathematical equations. 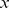 denotes protein peak with m/z value of 7787, 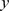 denotes protein peak with m/z value of 6858, and 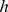 denotes protein peak with m/z value of 6646. Define the recurrence function as
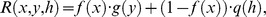
where

**Table 3:** Performance of the decision classification tree in predicting recurrence of HCC after MWA.

Sets	Recurrence	Percentage correct	Percentage misclassified
Training set	Yes (14)	85.7%(12/14)	14.3%(2/14)
	No (36)	88.9%(32/36)	11.1%(4/36)
Test set	Yes (10)	80.0%(8/10)	20.0%(2/10)
	No (26)	88.5%(23/26)	11.5%(3/26)



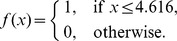


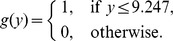


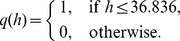



HCC will be recurrent if 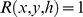, and HCC will not be recurrent if 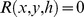.

### Validation of the Decision Tree

To evaluate the classification performance of the decision tree, we determined the sensitivity and specificity of the algorithm that was constructed with the training set, using the remaining 36 patients in the testing set. The decision classification tree correctly diagnosed 80.0% (8 of 10) patients with recurrence and 88.5% (23 of 26) patients without recurrence at 1 year after MWA (Table 3).

### Comparison of Decision Classification Tree and AFP Level for Early Recurrence Prediction

The sensitivity and specificity of decision classification tree and AFP to predict recurrence of HCC were examined in 86 HCC patients. A sensitivity of 88.3% (20 of 24) and specificity of 88.7% (55 of 62) for predicting early recurrence in HCC patients were obtained by decision classification tree analysis. In clinical practice, patients with AFP>20 ng/mL are considered to have liver abnormality. Therefore, this same AFP cutoff level was used in the current study to discriminate high-risk recurrent HCC patients. When AFP was used to predict HCC recurrence, a sensitivity of 54.2% (13 of 24) and specificity of 69.4% (43 of 62) were obtained. The diagnostic odds ratios (DOR) were also calculated in this study to compare the decision classification tree and AFP for predicting HCC in all samples. DOR is a single indicator for diagnostic performance, such that higher DOR value means better discriminative power. The calculated DOR of the decision classification tree was 41.50, implying that the odds for positivity among recurrent patients were 41.50 times higher than the odds for positivity among non-recurrent patients. The calculated DOR for AFP was only 2.68. Based on these values, decision classification tree performed 20 times better than AFP in predicting recurrence in HCC patients (41.50 *vs* 2.68) (Table 4).

**Table 4:** Sensitivity, specificity, and DOR of decision classification tree and AFP for HCC recurrence predicting.

Methords	Sensitivity (%)	Specificity (%)	DOR misclassified
Decision classification tree	88.3	88.7	41.5
AFP	54.2	69.4	2.68

## Discussion

Microwave ablation, which is developed as a minimally invasive thermal therapy for HCC, has been performed as a reliable alternative to hepatic resection in patients with small HCC [5]. In recent years, increasing numbers of patients have preferred to undergo microwave ablation rather than hepatic resection, and satisfied curative effect has been acquired in our institution [21]. Microwave ablation is a local ablation method, and local regrowth could be seen in some patients. Taking advantage of the tremendous progress of the technique [22] and the development of monitoring methods [23], such as CEUS [24], [25] and CE-MRI, the number of local residual tumors has been decreased dramatically. Promising results of microwave ablation for HCC have been demonstrated in many other studies [26]–[28]. In our study, follow-up after 1 year showed complete ablation in all patients studied. However, despite complete ablation of the tumor, recurrence in other segments of liver is still a problem in MWA treatment. The same problem also exists in surgical resection, the traditional treatment of HCC, and liver transplantation [29]–[31]. Early recurrence after surgery is closely correlated with poor prognosis of HCC [2]. Thus, there is an urgent need for proper markers to predict HCC recurrence in the early stage.

Proteomic analysis of sera and liver tissues from patients with HCC is an emerging technique for the identification of biomarkers indicative of disease severity and progression [32]–[34]. Analysis of serum or other body fluid that is easy to obtain from patients to predict disease or evaluate treatment efficacy would be ideal. Since abundant biological information exists in blood, characterization of serum proteome has become an essential step towards improvements in care for patients with HCC. The development of mass spectrometry proteomic profiling approaches, such as SELDI, allows greater sample throughput and the ability to examine low mass proteins. The major goal for most proteomic studies in HCC patients is the identification of markers for early detection of HCC [35]–[38]. In recent years, early detection and treatment of HCC have undergone great progress, but tumor recurrence remains a significant cause of death in patients with HCC.

In this study, our goal was not to identify patients with HCC, but rather to build a model that predicts clinical behavior in patients already diagnosed with HCC. The pathological state of the liver may be reflected by the serum proteome of an individual. Because HCC is derived from hepatocytes, these cells might release cellular proteins into circulation, including proteins that might function as biomarkers in predicting recurrence, or allowing early detection of recurrence in HCC patients. We hypothesized that biomarkers are present in preoperative serum, which might be used to predict the probability of recurrence following MWA. We used the SELDI ProteinChip system to analyze serum samples from patients with HCC. We tried to find a differentially expressed protein peak in recurrent patients and non-recurrent patients. Beyond our expectations, nine protein peaks within an m/z range of 1,000 to 30,000 differed significantly between recurrent patients and non-recurrent patients. Based on the peak intensities of the 87 peak proteins, three peaks were selected to construct a decision classification tree using Biomarker Patterns Software. The decision classification tree was proposed for screening of patients at high risk of recurrence from all patients with HCC after MWA in 1 year. On the basis of the presence of the three protein peaks, the sensitivity and specificity of the decision classification tree were 85.7% and 88.9%, respectively.

Clinical proteomics is a non-targeted approach that allows the characterization of the whole, or part of the whole, spectrum of proteins in a biological sample. Therefore, proteomics approaches have been already applied to the diagnosis of HCC. An accurate prognosis is essential, particularly in malignant diseases. The ability to accurately predict early recurrence, especially if it could be done well in advance of its occurrence, could provide advice to patients and guidance for assessment and treatment that could most benefit patients, with the goal of delaying recurrence and prolonging survival. At present, there is no widely accepted tool for predicting recurrence after MWA in HCC patients. If a sensible predictor of prognosis could be applied to patients with HCC, particularly with early stage HCC, it would be helpful in choosing rational treatment decisions. The present study identifies preablation serum SELDI-TOF MS profiling features that are useful in investigating the risk of recurrence, and the decision classification tree could predict the probability of developing recurrent HCC with an accuracy of 86.1% (31/36). Importantly, follow-up should be focused on patients who are identified as being at high risk for recurrent HCC after MWA, and who might be candidates for adjuvant therapy, such as immune-mediated therapy in the future. However, further studies with larger cohorts are needed to verify the decision classification tree gradually. The decision classification tree combined with serum candidate proteins is a potential tool in predicting early intrahepatic recurrence of HCC.

In conclusion, this study demonstrated that preablation serum proteomic analysis is a significant indicator for predicting early recurrence in HCC patients after MWA. Preablation serum proteomic profiling should help to identify patients who may benefit from more intensive surveillance after MWA and additional therapies before the appearance of recurrence.
